# A "Return" Case of Scarlet Fever

**Published:** 1904-05-07

**Authors:** 


### A "Return" Case of Scarlet Fever.

The case of Wright *versus* The Managers of the Metropolitan Asylums District recently tried in the High Court is one of considerable public and professional interest. It was alleged on the part of the plaintiff that one of his children who had been suffering from scarlet fever was discharged from the Grove Hospital, Tooting, whilst still capable of conveying infection, and that as a result several other members of the family acquired the disease. On this was founded a charge of negligence against the hospital authorities, and the Asylums Board was sued as responsible for the acts of their agents. It was admitted that the child was discharged from the hospital on a date five weeks and four days after admission, and that the mother and two other children shortly afterwards were attacked with scarlet fever; and there was no attempt to question the suggestion that the first case was responsible for the later developments. The plaintiff, however, alleged that when the boy came home he was suffering from a nasal discharge, and on the question of fact this formed the central point of the case. According to the hospital record there had been during the child's residence some suspicion of the presence of rhinitis, but there was no nasal discharge when he left the hospital. The nasal discharge, it was contended by the defendants, was a later development and was probably due to exposure of the boy to an east wind when he was taken home on the top of an omnibus. It was not denied that this development was probably the cause of the later cases, but in view of the condition of the boy when he was sent home the hospital authorities denied the charge of negligence, and the Asylums Board consequently repudiated liability. At the trial the jury, after hearing the evidence of the medical superintendent of the Grove Hospital, stopped the case and gave a verdict for the defendants on the ground that negligence had not been proved, and with this view the judge concurred. Such a result must be satisfactory not only to the medical men immediately concerned but also to the profession generally. It is in harmony with the general principle of law which determines the limits of professional responsibility and which measures this not merely by consequences but by a fair standard of ability and attention. To the experienced it is a matter of common knowledge that the definition of a precise limit by means of which freedom from the ability to convey infection after an attack of one of the zymotic diseases is, in the present state of our knowledge, impossible. All that can be obtained is a somewhat rough approximation based on empirical observation, and this is found by experience to be accurate in the enormous majority of cases. Exceptional instances in which the virus or germs responsible for the disease remain in a latent condition, and are aroused into activity by subsequent events, will doubtless continue to occur, but to hold the profession responsible for the non-recognition of these before the event would be decidedly unjust. At the same time it is highly desirable both on scientific and social grounds that this small minority shall be still further diminished. It is in this respect highly satisfactory to note that all the medical men called as witnesses in the recent trial repudiated the suggestion that the selection of the date at which a scarlet fever convalescent may be allowed with safety to mingle with the public can be fixed by a mere mechanical calculation. The crucial point is not the lapse of six weeks or of any other period of time, but the state of the patient as critically determined by an expert medical observer. This is the sound and scientific basis on which to rest the responsibility of the decision, and it has the additional advantage of emphasising the necessity for a careful survey of all the facts in each individual instance. Such a survey is the best protection of the public against the premature discharge of any single patient, and is the only method by which a more exact knowledge of the conditions which determine infectivity can be accumulated. The public has a right to expect the most careful and exact application of the principles and methods which experience enforces, but when some exceptional instance shows that these principles and methods are not absolute it would be unfair to penalise the individual practitioner. It is the duty of the medical profession to use every endeavour to increase its capacity for the protection of the public against the spread of epidemic disease, but it cannot act in advance of knowledge. And even when knowledge is perfect its application by human agents must at times be fallible. For those who suffer as the result of these possible mischances we must have every sympathy, and it might fairly be considered whether, when such suffering occurs as a consequence of an action taken for the public interest, the victims should not receive some measure of compensation.

